# Comparative analysis of management practices and end-users’ desired breeding traits in the miracle plant [*Synsepalum dulcificum* (Schumach & Thonn.) Daniell] across ecological zones and sociolinguistic groups in West Africa

**DOI:** 10.1186/s13002-021-00467-8

**Published:** 2021-06-19

**Authors:** Dèdéou A. Tchokponhoué, Enoch G. Achigan-Dako, Sognigbé N’Danikou, Daniel Nyadanu, Rémi Kahane, Alfred O. Odindo, Julia Sibiya

**Affiliations:** 1 School of Agricultural, Earth and Environmental Sciences, University of KwaZulu-Natal, Private Bag X01, Scottsville, Pietermaritzburg, 3209 South Africa; 2 Laboratory of Genetics, Biotechnology and Seed Science (GBioS), School of Plant Sciences, University of Abomey-Calavi, 01 BP 526, Abomey-Calavi, Republic of Benin; 3 World Vegetable Center, East and Southern Africa, Po. Box 10, Duluti, Arusha, Tanzania; 4 Cocoa Research Institute of Ghana (CRIG), P. O. Box 8, Akim Tafo, Ghana; 5 Research Unit HortSys, Department Persyst, CIRAD, Campus de Baillarguet, 34398 Montpellier cedex 5, France

**Keywords:** Ecological zones, Ethnicity, Orphan crops, *Richardella dulcifica*, Trait preference

## Abstract

**Background:**

Understanding end-users’ preferred breeding traits and plant management practices is fundamental in defining sound breeding objectives and implementing a successful plant improvement programme. Since such knowledge is lacking for *Synsepalum dulcificum*, a worldwide promising orphan fruit tree species, we assessed the interrelationships among socio-demography, ecology, management practices, diversity and ranking of desired breeding traits by end-users of the species (farmers, final consumers and processing companies) in West Africa.

**Methods:**

Semi-structured interviews, field-visits and focus groups were combined to interview a total of 300 farmers and final consumers belonging to six sociolinguistic groups sampled from three ecological zones of Benin and Ghana. One processing company in Ghana was also involved. Data collected included socio-demographic characteristics; crop management systems and practices; and preferences of farmers, final consumers and processing companies and ranking of breeding traits. Data were analysed using descriptive statistics, independence, and non-parametric tests, generalized linear models, multi-group similarity index and Kendall’s concordance coefficient.

**Results:**

Men (86.33% of respondents) were the main holders of *S. dulcificum* in the study area. The three most frequent management practices observed in the species included weeding, fertilization and pruning, which were applied by 75.66%, 27.33% and 16.66% of respondents, respectively. The management intensity index varied significantly across ecological zones, sociolinguistic groups, and instruction level (*p* < 0.001) but was not affected by gender (*p* > 0.05). General multigroup similarity indices (${\mathrm{C}}_{\mathrm{S}}^{\mathrm{T}}$) for farmer-desired traits, on one hand, and final consumer-desired traits, on the other hand, were high across ecological zones (${\mathrm{C}}_{\mathrm{S}}^{\mathrm{T}}$ ≥ 0.84) and sociolinguistic groups (${\mathrm{C}}_{\mathrm{S}}^{\mathrm{T}}$ > 0.83). Nevertheless, respondents from the Guineo-Congolian (Benin) and the Deciduous forest (Ghana) zones expressed higher agreement in the ranking of desired breeding traits. Preference for breeding traits was 60% similar among farmers, final consumers, and processors. The key breeding traits desired by these end-users included in descending order of importance big fruit size, early fruiting, high fruit yielding (for farmers); big fruit size, high fruit miraculin content, fruit freshness (for final consumers); and high fruit miraculin content, big fruit size, high fruit edible ratio (for processing companies).

**Conclusion:**

This study revealed stronger variations in current management practices across ecological zones than across sociolinguistic groups. A high similarity was shown in end-users’ preferences for breeding traits across the study area. Top key traits to consider in breeding varieties of *S. dulcificum* to meet various end-users’ expectations in West Africa include fruit size and fruit miraculin content. These results constitute a strong signal for a region-wide promotion of the resource.

**Supplementary Information:**

The online version contains supplementary material available at 10.1186/s13002-021-00467-8.

## Background

**Fig. 1:**
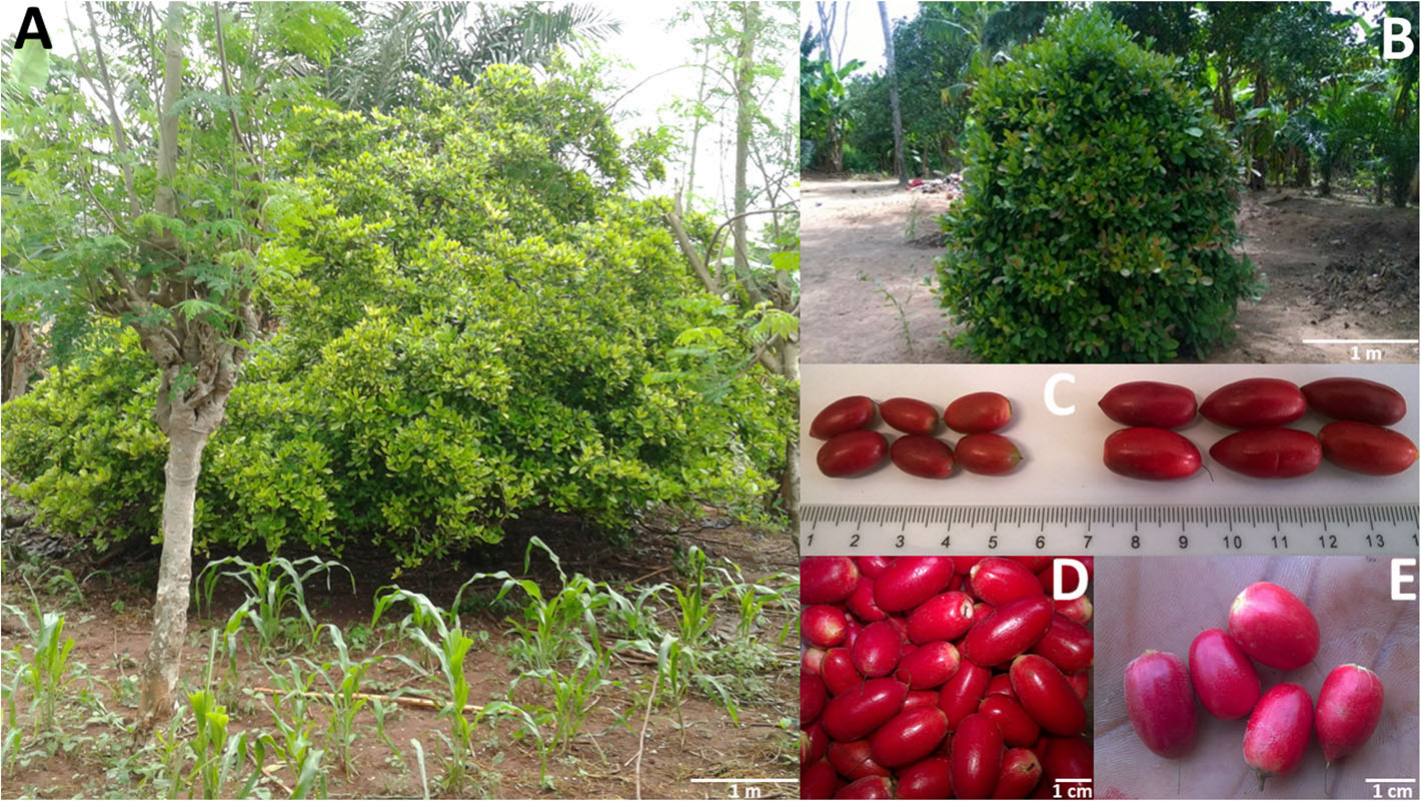
Traits variation in *Synsepalum dulcificum*. Tree size variation (**A-B**); Fruit size variation (**C**) and fruit shape (**D**: oblong fruit, **E**: Ovoid fruit) variation

*Synsepalum dulcificum* is a slow-growing and long-living West African native fruit tree species belonging to the Sapotaceae family. The species can grow up to a height of 7.5 m at maturity (120 years old) with a crown diameter ranging from 0.75 to 8.8 m (Fig. 1A, B) [1]. Its mature and ripe fruits are 7–18-mm-wide and 13–26-mm-long oblong to ovoid-shaped red berries (Fig. 1C–E) [1] named “miracle berry”. The fruit is on average 10.5 mm wide and 18.8 mm long, whereas the tree is on average 3.4 m tall. The species is thought to combine both autogamy and allogamy though the preponderant mating system is yet to be determined [2]. *Synsepalum dulcificum* is mainly propagated by seeds, which are recalcitrant [3, 4]. Alternative propagation methods such as cutting and layering are not commonly used because of the difficult adventitious rooting in the species.

Globally classified as a Least Concern species (Link 1), *S. dulcificum* is threatened and endangered in West Africa [1, 5, 6], its centre of origin and diversity, but is considered as one of the most valuable neglected berry crops because of its potential worldwide. The miracle berry is a unique natural source of miraculin, a sweetening glycoprotein that changes sour taste to sweet [7] and serves as a natural sweetener in the food and beverage industries [8, 9]. It is also used in diabetes and cancer treatments [10] as well as in cosmetics for the treatment of hair breakage [11]. However, *S. dulcificum* remained poorly documented from many points of view; for instance, information related to its management practices, production constraints and trade chain does not exist. Internationally, the miracle berry fetches a high price (over USD 2500/kg) (Link 2, Link 3), but income generation from the species by farmers at the grass-root level is yet to be properly documented. Similarly, knowledge on end-users’ preferred traits, though fundamental to breeding in the species, is still lacking. A thorough characterization of the species recently conducted in Benin, Togo and Ghana revealed three phenotypic groups discriminated by the combined tree and fruit traits, which suggested a significant trait variation in the species [1]. Specifically, it was established that *S. dulcificum* trees in Ghana (wetter region) produced longer, larger and heavier fruits than trees sampled in Benin (drier region) [1]. However, the extent to which the local populations perceived such traits variation in general and whether they can recognize any variant/morphotype in the species are yet to be documented. Using random amplified polymorphic DNA markers, Chibuzor et al. [12] reported low genetic variation in Southern Nigerian populations of the species.

Participatory elicitation of breeding traits preference as a premix to implementing sound breeding programmes in plant species has been the centre of interest of many studies tackling a diversity of crop commodities including cereals [13, 14], legumes and pulses [15–17], vegetable, tuber and root crops [18–21]. Noticeably, the core of these works was on annual and bi-annual crops; and except the works of the World Agroforestry Centre in its effort to promote the domestication of indigenous fruit tree species [e.g. bush mango, *Irvingia gabonensis* (Aubry-Lecomte ex. O'Rorke) Baill.; safou tree, *Dacryodes edulis* (G. Don) HJ Lam; and vegetable tallow tree (*Allanblackia floribunda* Oliv.)] [22], studies on participatory breeding in African indigenous fruit tree crops are limited [23]. In addition, these participatory studies mostly focused on “farmers” and did not integrate other user target groups such as final consumers or even processors, whose preferences are equally important and could somehow affect the definition of breeding objectives. This focus on farmers only, consequently constitutes a limitation that has also been highlighted by Hussein [24] and Anja et al. [25]. These authors emphasized on the necessity to expand trait preference evaluation exercise to various user groups to decipher as much as possible diversity in preferences.

Trends in preference for breeding traits by farmers indicated the influence of numerous factors including ecological conditions and socio-demographic factors such as ethnicity, gender and landholding [17, 19, 26], among others. In Nigeria for instance, while women emphasized the processing traits (e.g. easy to peel) in cassava (*Manihot esculenta* Crantz), men were more interested in agronomic traits [27]. The same authors also reported that traits such as “early maturity” and “high yielding” were more frequently sought-after by farmers in Southeast Nigeria compared with those in the Southwest, whereas the reverse held true when it came to a trait such as “cooking time”. A comparison of preference for groundnut (*Arachis hypogaea* L.) revealed that while farmers in Ethiopia put a particular emphasis on traits such as early maturity, shell yield and drought resistance, those in Togo mainly targeted pod yield, pod size and oil yield [15, 28]. In contrast, a high similarity was observed between Benin and Togo farmers for trait preference in groundnut [15, 29]. In ackee (*Blighia sapida* K.D. Koenig), a minor multipurpose fruit tree species, preference for fruit-traits varied among Benin sociolinguistic groups; the *Batoonuu* group considered exclusively the fruit size, the *Natemba* group preferred the aril colour, while the *Otamari* rather indicated the aril size as their trait of interest [23]. Instances of sociolinguistic group-specific preferred breeding traits were also reported in Kersting's groundnut [*Macrotyloma geocarpum* (Harms) Maréchal and Baudet] [17], groundnut [29] and African locust bean (*Parkia biglobosa* Jacques) [30], among other crop species. It is also well studied that production constraints account for an important part in articulation and ranking of preferred breeding traits by farmers [31]. Consequently, the intensity of management practices defined as a set of actions directly or indirectly implemented by farmers to ensure the availability and the sustainability of plant production [32] is likely to shape farmers’ preference for breeding traits.

Building on the above-mentioned limitations, this study was undertaken to assess the interrelationships between socio-demographic factors, ecological conditions, management practices, diversity and preference for breeding traits of three different end-user groups of the miracle plant [*Synsepalum dulcificum* (Schumach & Thonn.)] in West Africa that included the farmers, the final consumers and the processing companies.

This study conducted in Ghana and Benin addressed the following questions: (i) What are the ongoing management practices in *S. dulcificum* and how are they influenced by socio-demographic factors and agro-ecological conditions? (ii) How diverse are end-users’ desired breeding traits in the species and to what extent are they influenced by socio-demographic variables and agro-ecological conditions? and (iii) How similar are desired breeding traits among farmers, final consumers and industrial processors? The answers to these questions will pave the way for defining sound breeding objectives to meet multiple actors’ preferences for the species in West Africa.

## Methods

### Study area

**Fig. 2:**
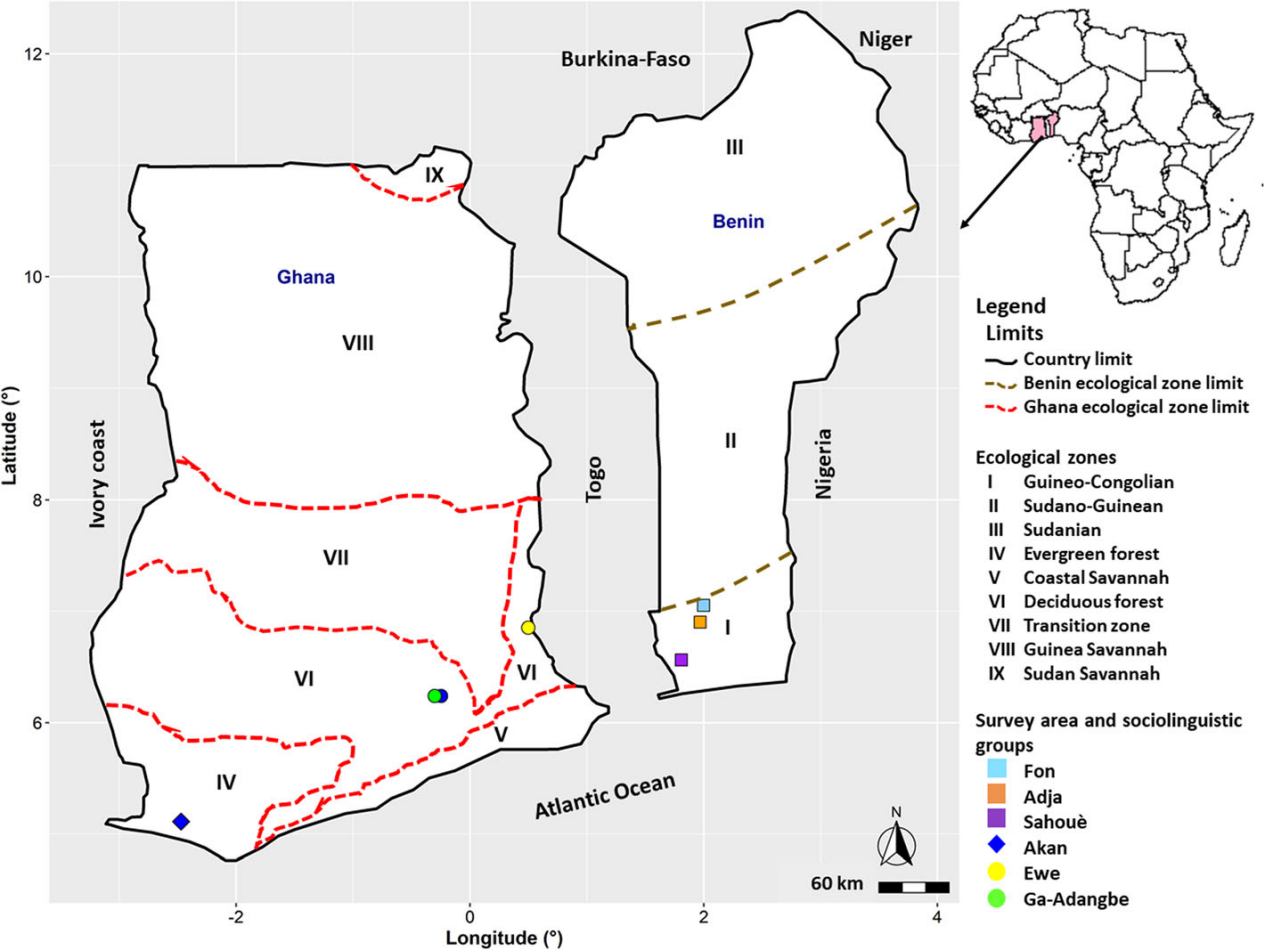
Map showing the study area

This study was conducted from April to December 2019 in Benin and Ghana (Fig. 2), two West African countries indicated as the centre of origin of *S. dulcificum* [2]. In Benin, *S. dulcificum* is confined to only one (Guineo-Congolian zone) out of the three ecological zones of the country, whereas in Ghana it is found in three (Evergreen forest, Deciduous forest and Transitional zones) out of the six ecological zones of the country. Six regions (three per country) were chosen based on the presence of the species. While in Benin all the three regions (Zou, Mono, Couffo) were part of the Guineo-Congolian ecological zone, those of Ghana (Volta region, Eastern region and Western region) belonged to two distinct ecological zones: the Evergreen ecological zone represented by the Western region, and the Deciduous forest ecological zone represented by the Eastern and Volta regions.

The Guineo-Congolian zone (GC) in Benin is characterized by a sub-equatorial climate with a bimodal rainfall pattern [33]. The rainfall ranges from 900 to 1300 mm with an average of 1200 mm while the annual temperature ranges between 25 and 39 °C with an average of 28 °C. The region experiences a relative humidity ranging between 69 and 97% [5, 34], and dominant soil types include ferralitic and ferruginous soils. The Deciduous forest ecological zone (DF) in Ghana also has a bimodal rainfall pattern with an annual rainfall varying from 1200 to 1600 mm with an average of 1500 mm [35]. The mean annual temperature in this ecological region is 26.4 °C and the soil is predominantly of eutric nitosol type [36]. In the Evergreen forest ecological zone (EF) of Ghana, the rainfall ranges from 800 to 2800 mm with an average of 2200 mm [35]. The mean annual temperature is 26.1 °C and predominant soil types are acrisols and ferralsols [35–37]. In terms of moisture gradient, EF is a moist zone, DF a moderately moist and GC a dry zone.

### Interview sampling method

#### Individual respondents (farmers and final consumers)

Six sociolinguistic groups (three in Benin and three in Ghana) were selected for this study. In Benin, these groups included the *Fon*, *Adja* and *Sahouè*, while in Ghana, the three groups were *Akan*, *Ga-adangbe* and *Ewe*. The *Ewe* and *Ga-adangbe* groups inhabit the Deciduous forest ecological zone, while the *Akan* group was found in both Deciduous and Evergreen forest ecological zones. The study focused on informants who had the species on their farms to gather information on management practices and preferences [38]. Consequently, we combined convenience sampling and snowball techniques, two commonly used non-probability sampling methods [39, 40], to select the respondents. Snowball was used to identify potential respondents who own and or cultivate the species. The final respondents then included those who (i) had at least 5 years’ experience in the management or cultivation of the species, (ii) had their trees already in reproductive phase (bearing fruits), (iii) were miracle berry-consumers and (iv) had formally given their consent to participate in the study after the aim of the study was explained to them. These filters were imposed to ensure that respondents fully addressed the research questions.

#### Industrial processing company

To identify *S. dulcificum* processing companies in Benin and Ghana, we conducted online research, crossed-checked with the information provided by owners/farmers. They were asked to list any company they traded their product with or were aware of. Internet search was conducted in the Google search engine with the following research terms: (1) “Miracle:berry / Sweet:berry / *Synsepalum dulcificum*; Company; Ghana / Benin”, (2) “Miracle:berry / Sweet:berry / *Synsepalum dulcificum* + Company + Ghana / Benin”, (3) “Miracle:berry / Sweet:berry / *Synsepalum dulcificum*:Company:Ghana / Benin”. The identified processing companies were contacted afterwards, and those who gave their consent were included in the study.

### Data collection

#### Individual respondents (farmers and final consumers)

Semi-structured interviews based on a questionnaire were combined with field visits to collect data on individual respondents. Interviews were conducted using the respondents’ preferred language, which was either Adja, Fon, or Sahouè in Benin; and Twi, Ewe, or Ga in Ghana. To facilitate the communication between interviewers and interviewees, each interviewer (where necessary) was accompanied by a well-trained local guide (who understood both the interviewee-spoken language and Fench/English) to facilitate the questions/answers translation. Direct interviews using either English or French were done, where necessary.

The data collected were related to (i) the socio-demographic background of the informants, (ii) *S. dulcificum* ownership (individual trees: miracle plant trees found isolated (not in a group) versus plantation: in which the miracle plant trees were found in a group ( > 100 trees in the context of this study) installed in a clear layout marked by a regular spacing among trees, (iii) the production system in which the species has evolved (home garden: a production system near dwellings, and somehow well controlled by the owner for the target products versus on-farm: an open agricultural production space usually farther away from the dwellings and larger than a home garden [1, 41]), (iv) the relative importance of *S. dulcificum* in the production system, (v) cropping practices *of S. dulcificum*, (vi) the informant’s awareness of grown *S. dulcificum* morphotypes and varieties, (vii) the farmers’ desired-traits for an improved variety of *S. dulcificum* and (viii) the final consumer-desired breeding traits for an improved variety of *S. dulcificum*. To characterize the production system in which *S. dulcificum* is involved and the relative importance of this latter in the whole system, *S. dulcificum*-based production systems were visited, and the top five most important species based on all possible functional attributes held (food, medicine, market value etc.) were elicited with the farmer. Then, the pairwise comparison technique was used to score each of the five species mentioned by the farmer on a scale ranging from 0 (the least important crop) to 5 (the most important one). The same listing of the five most important attributes and their subsequent scoring in a pairwise comparison scheme was also applied to (i) the farmer-desired traits for an improved variety of *S. dulcificum* and (ii) the final consumer-desired breeding trait for an improved variety.

The taxonomic identification of the species, indicated as important by the respondents, was first done in situ by the first author using available flora resources including the analytical flora of Benin [42], the trees, shrubs and lianas of West African dry zones manual [43], and the plant list (http://www.theplantlist.org/) online resource [44]. The identifications were afterwards confirmed through a formal taxonomic identification at the National Herbarium of Benin.

#### Industrial processing companies

A focus group interview was held with four members holding different positions in one processing company to collect information related to the company genesis, company production objectives and desired breeding traits for a variety suitable to each of the company’s production line as well as the pairwise ranking of these desired traits.

### Data analysis

The R environment version 3.6.2 [45] was used for data analyses.

Descriptive statistics (mean, standard error, range and frequency) were computed on the socio-demographic characteristics of respondents using the functions *descript ()* and *crosstab ()* of the package ‘misty’ [46]. The major plant species in the farmer’s production system, as well as the relative importance of *S. dulcificum* in the system, were assessed using descriptive statistics computed on scores obtained from the pairwise ranking. A χ^2^ test or Fisher exact test (to account for observation count < 5) was used to test dependence between *S. dulcificum* holding systems and categorical socio-demographic factors (gender, ethnicity, instruction levels) on one hand, and ecological zone on the other. To characterize the importance of management practices in the species, we followed the framework developed by Blancas et al. [47] to propose a new index, the boosted management intensity index (BMI), which is adapted from the management intensity index (IM) developed by Sogbohossou et al. [39]. This new index is based on ten key indicators of management practices (Table 1) and incorporates weighing coefficients to reflect better the situation in perennial species where certain practices are to be repeated over time. The coefficients are proposed to consider elapsed time since some management practices (e.g. pruning, weeding) were applied for the last time. For a specific respondent *j* the BMI is computed as:

$$ {BMI}_j=\sum \limits_{i=1}^m{IL}_i+\sum \limits_{i=1}^m{C}_i{ML}_i+\sum \limits_{i=1}^m{C}_i{UI}_i+ PSD+ RH+\sum \limits_{i=1}^m{UT}_i+ TH+ TI+ LF+ MF $$

**Table 1:** Variables used in the computation of the boosted management intensity index (BMI)

Variables	State of variables and codified scores	Weighing variable		Weighing coefficient
Installation labour (IL)	Land clearing (1); pegging and lining (1); holing (1); base manuring (1)	None	–	1
Management labour (ML)	Weeding (3); pruning (3); irrigation (3); fertilization (3)	Time since last application	≤ 1 year	1
Use of inputs (UI)	Do not use agrochemicals (0); use agrochemicals (3)		2–10 years	2/3
			> 10years	1/3
Distance to cultivation site (PSD)	≤ 100 m (1); up to 1 km (2); up to 5 km (3); more than 5 km (4)	None	_	1
Reaction to unauthorized harvest (RH)	No reaction (0); yes, admonition applies (1); yes, monetary sanction (2); yes, complain to authority (3)	None	–	1
Use of tools (UT)	Manual (1); hook (2); knife (3); machete (1)	None	–	1
Type of harvest (TH)	Opportunistic (1); planned (2)	None	–	1
Time invested (TI)	Min (1); hours (2); days (3)	None	–	1
Labour force invested (LF)	No staff hiring (1); staff hiring (2)	None	–	1
Management form (MF)	Collection from the wild (1); protection on farm (2); protection in home garden (3); seedling transplantation (4); seed sowing (5)	None	–	1

where *BMI*_*j*_ is the index computed for the respondent *j*, *m* is the total number of management practices constituting a specific management variable, *C*_*i*_ is the weighing coefficient associated to a management practice *i*, *IL*_*i*_ is the score for the practice *i* of the variable “establishment labour”, *ML*_*i*_ is the score for the practice *i* of the variable “management labour”, *UI*_*i*_ is the score related to the use of input, *PSD* is the score related to the distance to the cultivation/production site, *RH* is the score related to the reaction to an unauthorized harvest, *UT* is the score related to the use of tools in the species management, *TH* is the score related to the type of harvest, *TI* is the score related to the time invested in the species management, *LF* is the score related to the labour force invested in the species and *MF* is the score related to the management form.

The variation of BMI following ecological zone and socio-demographic factors was analysed using a generalized linear model with a Poisson or quasi-Poisson (to account for overdispersion) error structure. The effect of the holding system on income generation was analysed using a Wilcoxon test due to normality assumption violation. We used the *prop.test*-based binomial test to assess the difference in fruit production objective following ecological zone and sociolinguistic group. The similarity of farmer’s and final consumer’s desired breeding traits among ecological zones, gender, and sociolinguistic groups on one hand, and the similarity of traits preference among farmers, final consumers and processors on the other were analysed using the general multi-group similarity index (${\mathrm{C}}_{\mathrm{S}}^{\mathrm{T}}$) computed following Diserud and Ødegaard [11] as:

$$ {\mathrm{C}}_{\mathrm{S}}^{\mathrm{T}}=\frac{T}{T-1}\left(\frac{\sum \limits_{i<j}{a}_{ij}-\sum \limits_{i<j<k}{a}_{ij k}+\sum \limits_{i<j<k<l}{a}_{ij k l}-\sum \limits_{i<j<k<l<m}{a}_{ij k l m}+\dots }{\sum \limits_i{a}_i}\right) $$

where *T* is the total number of groups for which the index is computed, *a*_*i*_ is the number of traits listed by the group *A*_*i*_, i =1, 2, 3,…,T; *a*_*ij*_ is the number of traits shared by groups *A*_*i*_ and *A*_*j*_; *a*_*ijk*_ is the number of traits shared by *A*_*i*_, *A*_*j*_, *A*_*k*_ etc.

${\mathrm{C}}_{\mathrm{S}}^{\mathrm{T}}$ value ranges from 0 (no similarity in traits preference) to 1 (total similarity in traits preference). An illustration of the calculation of this index is provided in Table 2.

**Table 2:** Calculation of the general similarity index for more than two groups

Let case 1 be a study comparing traits preference in a species “A” by farmers from three different socio-cultural groups (1, 2 and 3) and whose preferred traits are elicited as follows: **A1**: [a, b, c, d, e]; **A2**: [a, d, e, f, h] and **A3**: [b, g, h, j]. The objective is to calculate the general multi-group similarity index of preferred traits among these three socio-cultural groups. For this example, T = 3, a_1_ = 5, a_2_ = 5 and a_3_ = 4, a_12_ = 3, a_13_ = 1, a_23_ = 1, a_123_ = 0 ${\mathbf{C}}_{\mathbf{S}}^{\mathbf{3}}$**=**$\frac{T}{T-1}\ \left(\frac{\sum \limits_{\boldsymbol{i}<\boldsymbol{j}}{\boldsymbol{a}}_{\boldsymbol{i}\boldsymbol{j}}-\sum \limits_{\boldsymbol{i}<\boldsymbol{j}<\boldsymbol{k}}{\boldsymbol{a}}_{\boldsymbol{i}\boldsymbol{j}\boldsymbol{k}}}{\sum \limits_{\boldsymbol{i}}{\boldsymbol{a}}_{\boldsymbol{i}}}\right)$ = $\frac{3}{3-1}\ \left(\frac{\left[{a}_{12}+{a}_{13}+{a}_{23}\ \right]-{a}_{123}}{a_1+{a}_2+{a}_3}\right)$= $\frac{3}{3-1}\ \left(\frac{\left[3+1+1\right]-0}{5+5+4}\right)$ ${\mathbf{C}}_{\mathbf{S}}^{\mathbf{3}}=\frac{3}{2}\left(\frac{5}{14}\right)$ ${\mathbf{C}}_{\mathbf{S}}^{\mathbf{3}}$**=**$\frac{15}{28}$ The similarity index of preferred traits among these three socio-cultural groups is 0.53, which reflects a moderate preference similarity.	

The agreement in the ranking of farmer-desired breeding traits across ecological zones and across sociolinguistic groups was assessed with the Kendall-W coefficient of concordance computed using the function *Kendall ()* of the ‘irr’ package [48]. The same coefficient was also computed in the case of final consumer-desired traits and to analyse the concordance in the ranking pattern of desired traits by the three user groups (farmers, final consumers, and processors) in this study.

## Results

### Socio-demographic characteristics of respondents

A total of 300 individual respondents were interviewed in this study. The distribution of the sampling size across ecological zones and sociolinguistic groups as well as the socio-demographic background of these respondents are detailed in Supplementary Table S1, Additional file 1. The proportion of women (13.67%) involved in *S. dulcificum* management was significantly lower than that of men (86.33%) (χ^2^ = 313.9, df =1, *p* < 0.0001). The goodness of fit test indicated that this ratio 14:86 of women/men holding the species was statistically constant across sociolinguistic groups (χ^2^ ≤ 0.96, df = 1, *p* > 0.05) and ecological zones (χ^2^ ≤ 0.39, df = 1, *p* > 0.05). The holders of *S. dulcificum* were mainly autochthons (χ^2^ = 420.01, df = 1, *p* < 0.0001) with 67% of them educated; and approximately 40% of them reached the level of secondary school (χ^2^ = 189.16, df = 4, *p* < 0.0001). Almost all respondents were married. The youngest respondent was 25 years old, an *Adja*, whereas the oldest was 102 years old from the *Ga-adangbe* sociolinguistic group. On average, the respondents were 55.47 ± 0.91 years old. Household size ranged from 2 to 30 with an average of 7.25 ± 0.24 members per household and differed significantly across ecological zones (*p* < 0.0001) and sociolinguistic groups (*p* < 0.0001).

### Profile of miracle berry processing companies

Two companies, based in Ghana, namely “Sweet Life Group Ghana Limited” and “Miracle Fruit Processing Ghana Limited” invested in miracle berry processing in the study area. Miracle Fruit Processing Ghana Ltd, formerly known as MB Group Ghana and created in 2015, is well known by farmers (100% of plantation owners) in Ghana. On the contrary, no farmer mentioned the Sweet Life Group Ghana Ltd company, which is still operating. This latter company declined to participate in the study; so, only information from one company is captured in this study.

### *Synsepalum dulcificum* holding systems

**Fig. 3:**
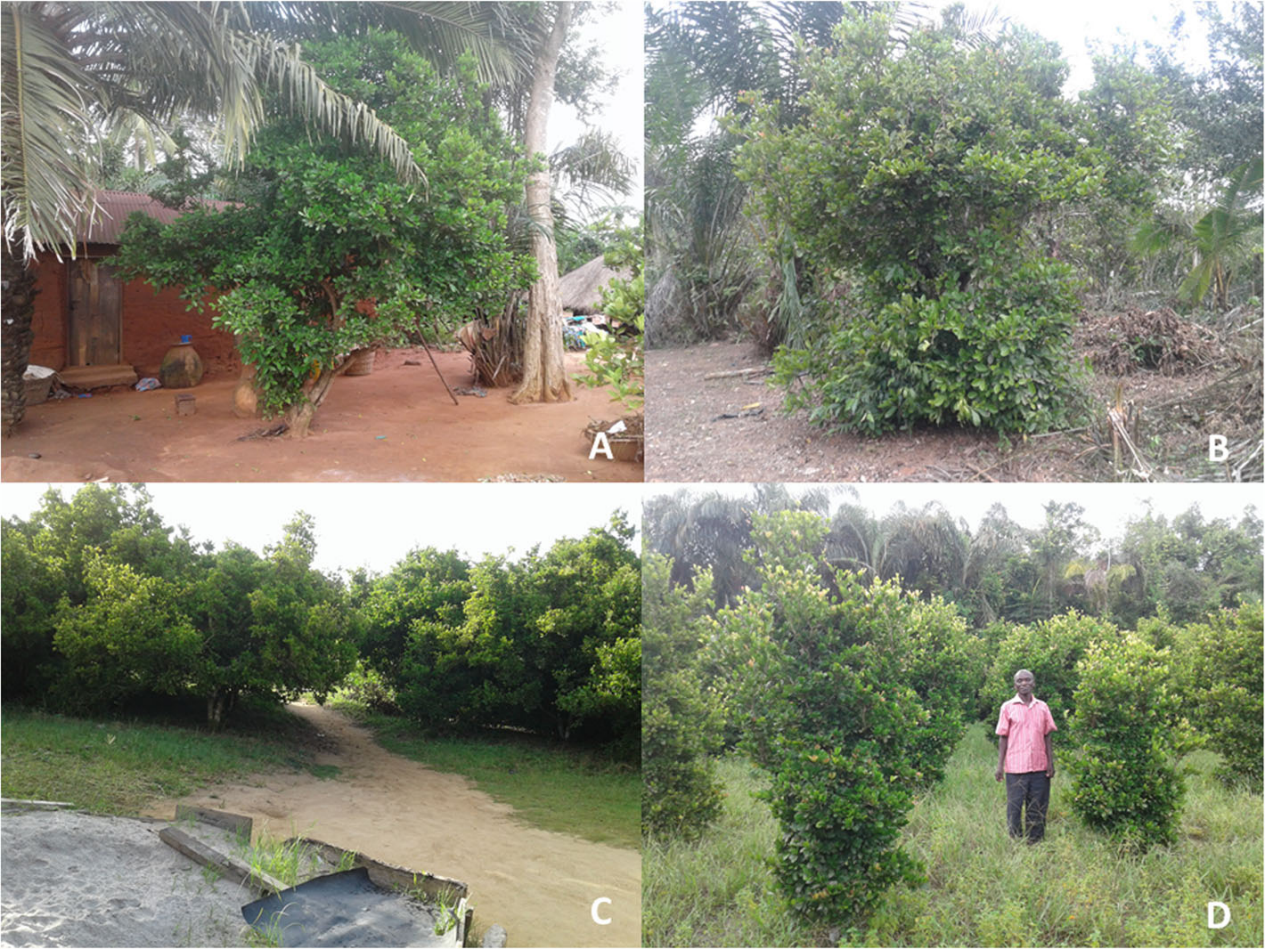
*Synsepalum dulcificum* in various production systems

Across the three ecological zones, the miracle plant was found in two production systems, namely home garden, and on-farm (Fig. 3A–D). While in the Guineo-Congolian zone the species was exclusively observed as individual trees (Fig. 3A), in the Deciduous and Evergreen forest zones it was present as either individual trees (Fig. 3B) and/or a commercial plantation (Fig. 3C, D).

Owners of individual trees of *S. dulcificum* represented 97.66% of respondents. The total number of individual trees they owned varied from 1 to 36 with an average of 2.79 ± 0.21 trees. Gender did not affect the number of individual trees of *S. dulcificum* owned (*p* = 0.427). In contrast, this number differed significantly among sociolinguistic groups (*p* = 0.006), with the *Ga-adangbe* (4.57 ± 0.64 trees) and the *Ewe* (3.30 ± 0.54 trees) owning two-fold more trees than the *Fon* (1.60 ± 0.12 trees). In parallel, respondents from the Deciduous forest zone (3.41 ± 0.32 trees) owned more trees than those from the Guineo-Congolian zone (1.84 ± 0.32 trees) (*p* < 0.001).

Within the Deciduous forest and Evergreen forest ecological zones, the main holding system depended on the sociolinguistic group affiliation (Fisher exact, *p* = 0.001), with the *Akan* being the only group owning plantations of *S. dulcificum*. Likewise, there was a significant association between instruction level and miracle plant holding system (Fisher exact, *p* = 0.002), and only educated respondents held plantations of *S. dulcificum*. Though only men owned *S. dulcificum* plantations, the test of independence indicated no significant association between gender and holding system (Fisher exact, *p* = 0.59). We recorded a total of 17 plantations in this study. The total number of plantations held per owner varied from 1 to 4 and with an average of 2.47 ± 0.36 plantations per owner. The size of a *S. dulcificum* plantation ranged from 0.16 to 89.03 ha in the study area, whereas the per-farmer cumulated acreage of *S. dulcificum* plantations ranged from 0.60 to 109.26 ha, out of which only 0–8.09 ha (representing approximately 8% increase relatively to the existing plantation acreage before 2015) were installed following the advent of the miracle fruit processing company, which indicated that most of the plantations were established before the company arrival. This illustrated that the company arrival did not boost or has not impacted yet the species plantation expansion.

### *Synsepalum dulcificum* relative importance in production systems

A total of 62 plant species from 32 families (Supplementary Table S2, Additional file 1) were recorded in the respondents’ production systems across the three ecological zones. Eighteen and 24 out of these 62 species had citation frequencies higher than 5% in home garden and on-farm production systems, respectively. The most important species for the respondents (Table 3) were significantly dominated by perennials in the home garden system (χ^2^ = 9.00, df = 1, *p* = 0.002), whereas annual species were as represented as perennial species in the on-farm production system (χ^2^ = 2.08, df = 1, *p* = 0.14). In general, *S. dulcificum* appeared poorly ranked in respondents’ production system, though it was perceived as more important in the home gardens than on-farm. Nevertheless, a disaggregated analysis indicated that *S. dulcificum* importance was differentially perceived across ecological zones and sociolinguistic groups. In the Evergreen forest ecological zone, *S. dulcificum* ranked 2nd and 3rd in home garden and on-farm production systems, respectively, whereas in Deciduous forest and Guineo-Congolian zones it was not in the top five species (Table 4). Similarly, the species was more integrated into *Akan*’s and *Sahouè*’s production systems than in any other sociolinguistic group (see Additional file 2). In contrast, both men and women ranked *S. dulcificum* out of the top 5 most important species (Supplementary Table S3, Additional file 1). Most important species included cocoa (*Theobroma cacao* L.), maize (*Zea mays* L.), oil palm (*Elaeis guineensis* Jacq.) and cassava (*Manihot esculenta* Crantz)*.*

**Table 3:** Diversity and relative importance of frequently involved species in *Synsepalum dulcificum*-based production systems

Species	On-farm		Species	Home garden	
	Importance score	Rank		Importance score	Rank
***Theobroma cacao*** **L.**	4.06	1st	***Theobroma cacao*** **L.**	4.39	1st
*Zea mays* L.	4.00	2nd	***Elaeis guineensis*** **Jacq.**	3.61	2nd
***Elaeis guineensis*** **Jacq.**	3.46	3rd	***Musa parasidica*** **L.**	3.55	3rd
*Manihot esculenta* Crantz.	3.30	4th	***Coffea canephora*** **L.**	3.43	4th
***Cola nitida*** **(Vent.) Schott & Endl.**	3.09	5th	*Manihot esculenta* Crantz.	3.27	5th
*Solanum aethiopicum* L.	3.00	6th	***Musa sapientum*** **L.**	3.12	6th
***Musa sapientum *L**.	2.55	7th	*Colocasia esculenta* (L.) Schott	3.00	7th
***Citrus sinensis*** **(L.) Osbeck**	2.40	8th	*Discorea alata* L.	3.00	8th
*Capsicum* sp.	2.31	9th	***Chrysophyllum albidum*** **G.Don**	2.79	9th
*Vigna unguiculata* (L.) Walp	2.29	10th	***Cocos nucifera*** **L.**	2.78	10th
***Tectona grandis*** **L.f.**	2.23	11th	***Citrus lemon*** **L.**	2.66	11th
***Irvingia gabonensis*** **(Aubry-Lecomte ex O'Rorke)**	2.19	12th	***Citrus sinensis*** **(L.) Osbeck**	2.66	12th
*Dioscorea alata* L.	2.18	13th	***Moringa oleifera*** **(Gaetn.) Dunal**	2.42	13th
***Musa parasidica*** **L.**	2.12	14th	***Annona muricata*** **L.**	2.38	14th
***Acacia auriculiformis*** **Benth.**	2.00	15th	***Mangifera indica*** **L.**	2.25	15th
***Annona muricata*** **L.**	2.00	15th	***Persea americana*** **Mill.**	2.22	16th
***Chrysophyllum albidum*** **G.Don**	2.00	15th	***Synsepalum dulcificum*** **(Schumach &Thonn.) Daniell**	1.70	17th
***Xylopia aethiopica*** **(Dunal) A. Rich.**	2.00	15th	*Ananas comosus* (L.) Merr.	1.28	18th
*Colocasia esculenta* (L.) Schott	1.94	19th	–	–	–
***Persea americana*** Mill.	1.87	20th	–	–	–
***Synsepalum dulcificum*** **(Schumach &Thonn.) Daniell**	1.83	21st	–	–	–
*Lycopersicum esculentum* L.	1.80	22nd	–	–	–
*Arachis hypogaea* L.	1.64	23rd	–	–	–
***Cocos nucifera*** **L.**	1.45	24th	–	–	–

Perennial species are in bold

**Table 4:** Per ecological zone- disaggregated importance of species involved in *Synsepalum dulcificum*-based production systems

Production system	Guineo-Congolian zone			Deciduous forest zone			Evergreen forest zone		
	Species	IS	Rank	Species	IS	Rank	Species	IS	Rank
On-farm	*Zea mays* L*.*	4.40	1st	*Theobroma cacao* L.	4.10	1st	*Manihot esculenta* Crantz.	5.00	1st
	*Elaeis guineensis* Jacq.	3.73	2nd	*Manihot esculenta* Crantz.	3.58	2nd	*Hevea brasiliensis* (Willd. Ex A. Juss.) Mull.Arg.	3.50	2nd
	*Manihot esculenta* Crantz.	2.96	3rd	*Zea mays* L*.*	3.42	3rd	*Synsepalum dulcificum* (Schumach &Thonn.) Daniell	3.40	3rd
	*Capsicum* sp.	2.42	4th	*Cola nitida* (Vent.) Schott & Endl.	3.10	4th	*Psidium guajava* L.	3.00	4th
	*Citrus sinensis* L*.*	2.41	5th	*Solanum aethiopicum* L.	3.00	5th	*Theobroma cacao* L.	3.00	5th
Home garden	*Musa sapientum* L.	3.38	1st	*Theobroma cacao* L.	4.41	1st	*Musa sapientum* L.	5.00	1st
	*Elaeis guineensis* Jacq.	3.37	2nd	*Elaeis guineensis* Jacq.	3.73	2nd	*Synsepalum dulcificum* (Schumach &Thonn.) Daniell	4.70	2nd
	*Citrus lemon* L.	3.30	3rd	*Musa parasidica* L.	3.55	3rd	*Psidium guajava* L.	4.00	3rd
	*Citrus sinensis* (L.) Osbeck	3.12	4th	*Manihot esculenta* Crantz.	3.50	4th	*Musa parasidica* L.	3.50	4th
	*Cocos nucifera* L.	2.80	5th	*Coffea canephora* L.	3.43	5th	*Citrus sinensis* (L.) Osbeck	3.00	5th

*IS* importance score

### *Synsepalum dulcificum* management practices

#### Types and sources of planting materials

In the study area, respondents mainly used two types of planting material for the establishment of *S. dulcificum*: seeds, seedlings, or a combination of both. Overall, respondents used seedlings (55.21%) more frequently than seeds (44.79%) (χ^2^ = 4.60, df = 1, *p* = 0.03). While the choice of planting material depended neither on gender (χ^2^ = 1.20, df = 2, *p* = 0.55) nor on sociolinguistic group affiliation (χ^2^ = 15.37, df = 10, *p* = 0.14), we observed that inhabiting the Evergreen forest ecological zone strongly conditioned the use of seedlings as the only planting material (χ^2^ = 14.95, df = 4, *p* = 0.004)*.* Likewise, seeds were more frequently used in home-garden, and seedlings for on-farm production (χ^2^ = 2.60, df = 1, *p* = 0.08).

**Fig. 4:**
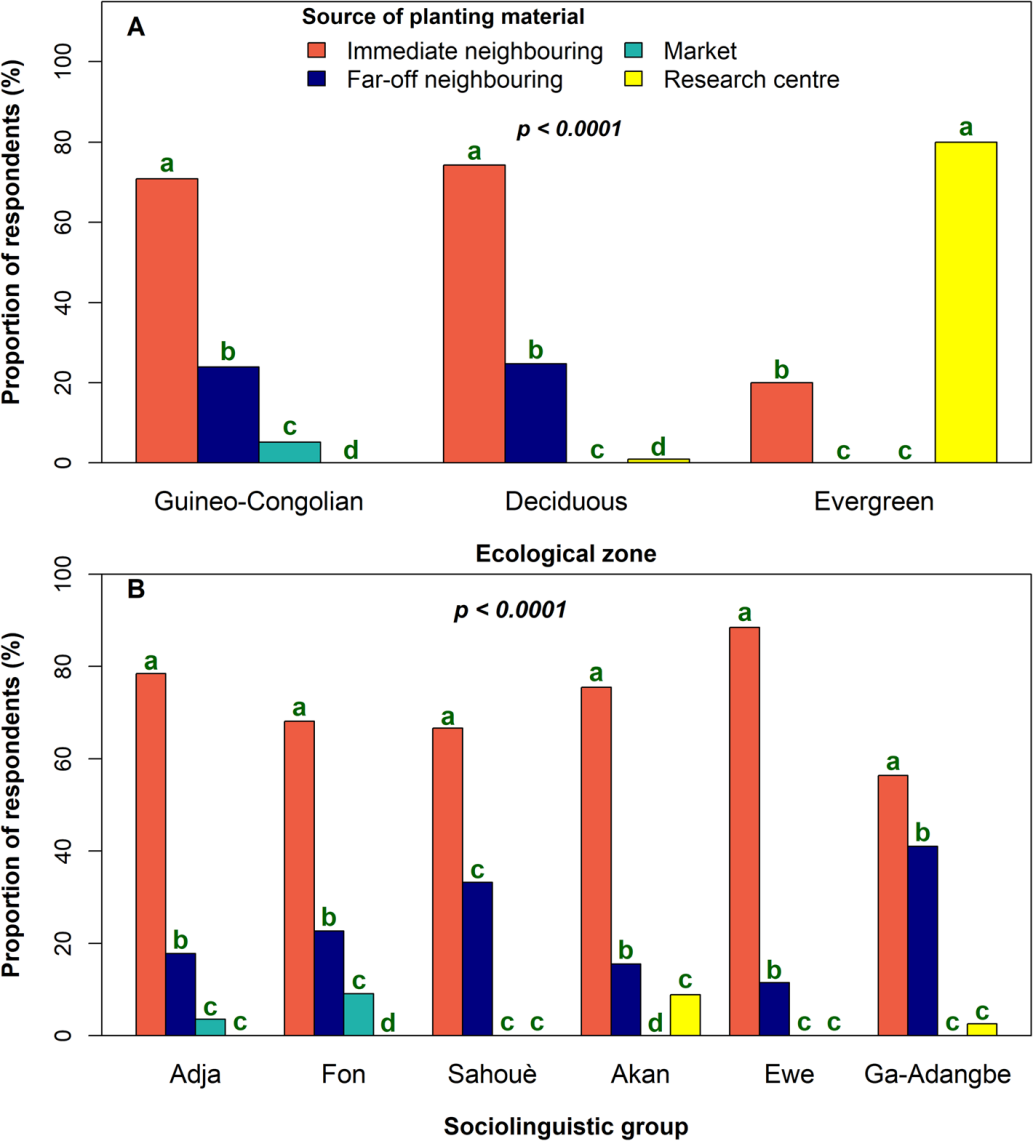
Relative importance of *Synsepalum dulcificum* planting materials’ source across ecological zones (**A**; Guineo-Congolian: Guineo-Congolian zone; Deciduous: Deciduous forest and Evergreen: Evergreen forest) and sociolinguistic groups (**B**)

These planting materials were obtained by farmers from four different sources including research centres, markets, immediate neighbour (sourcing neighbour located at < 5-km radius far from the respondent) and far-off neighbour (sourcing neighbour located at ≥ 5-km radius far from the respondent). Immediate neighbour was by far the commonest means of sourcing *S. dulcificum* planting material (χ^2^ = 174.29, df = 3, *p* < 0.0001). Seeds obtained from immediate neighbour or far-off neighbour were systematically sown without any clear selection criteria, while seedlings, which were not produced per se, but rather just uprooted beneath mother trees and transplanted were selected considering their vigour. The relative importance of each source disaggregated by ecological zone and sociolinguistic group is presented in Fig. 4. A research centre as source of planting material was only reported by respondents in Deciduous and Evergreen forest ecological zones, while purchase from the market only occurred in the Guineo-Congolian ecological zone. There, only the *Adja* and *Fon* respondents bought planting materials from the market, whereas in Ghana, only the *Akan* and *Ga-adangbe* accessed planting materials from a research centre. The *Ewe* and *Sahouè* respondents only accessed both seeds and seedlings from neighbours as gifts.

#### Variation of management intensity of *Synsepalum dulcificum*

**Fig. 5:**
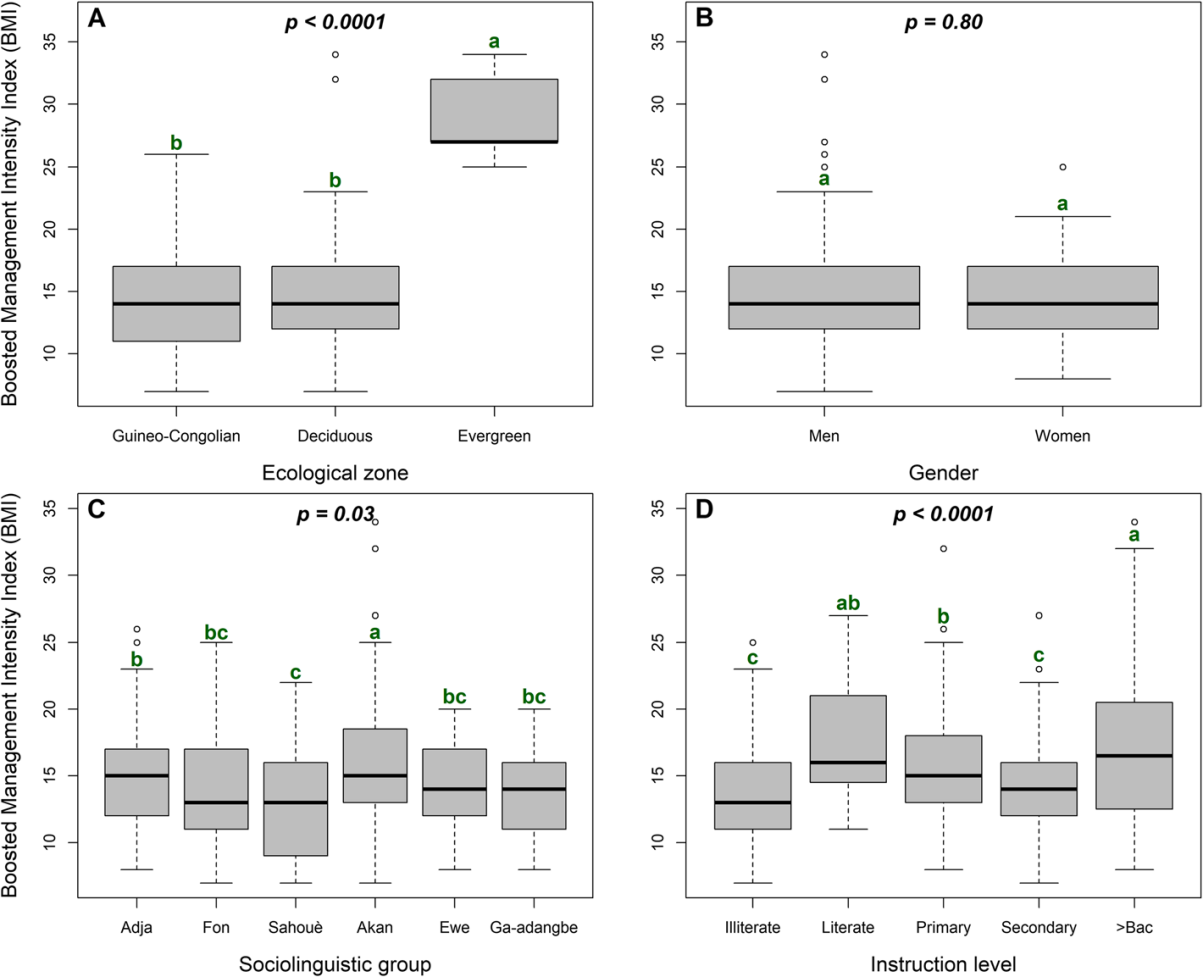
Variation of management intensity index in *Synsepalum dulcificum* following ecological zones (**A**; Guineo-Congolian: Guineo-Congolian zone; Deciduous: Deciduous forest and Evergreen: Evergreen forest), gender (**B**), sociolinguistic groups (**C**) and instruction levels (**D**)

The ten variables summarizing the set of management practices in *S. dulcificum* are indicated in Table 1. The combination of all these variables indicated that the boosted management intensity index (BMI) of *S. dulcificum* was on average 14.61 ± 0.24 and ranged from 7.00 to 34.00. This management intensity was significantly affected by the ecological zone and some socio-demographic factors. Management intensity index was two-fold higher in the Evergreen forest ecological zone (BMI = 29.0 ± 1.70) than in Deciduous forest (BMI = 14.54 ± 0.30) and Guineo-Congolian (BMI = 14.19 ± 0.32) zones (Fig. 5A), while the species management by women (BMI = 14.46 ± 0.57) was as intense as by men (BMI = 14.63 ± 0.26) (Fig. 5B). Regarding the influence of sociolinguistic groups, the *Akan* managed more intensely the species (BMI = 16.88 ± 0.86) than any other sociolinguistic groups, whereas the lowest management intensity index was obtained with the *Sahouè* sociolinguistic group (BMI = 13.26 ± 0.59) (Fig. 5C). Instruction level also exerted a significant effect on the management intensity (Fig. 5D) though increased instruction level did not necessarily reflect in higher management intensity. Plantations (BMI = 30.14 ± 1.14) were also better managed compared with individual trees (BMI = 14.24 ± 0.20) (*p* < 0.0001). There was no correlation between respondents’ age and management intensity (r = 0.02, *p* = 0.69) on one hand, and between experience in the species management and management intensity (r = − 0.03, *p* = 0.57) on the other.

#### Farmers’ awareness of morphotypes in *Synsepalum dulcificum*

For 100% of respondents, there is no variety of *S. dulcificum*. However, 6.33% of respondents indicated the existence of different morphotypes that they essentially distinguished through the difference in fruit size and fruit exocarp colour. Where morphotypes were reported, 100% of respondents differentiated big fruit sized from small fruit sized morphotypes, whereas only one out of the 300 respondents (from the Deciduous forest ecological zone of Ghana, and the *Ga-adangbe* sociolinguistic group) indicated the existence of a yellow morphotype in addition to the ordinary known red morphotype.

#### Farmer-desired breeding traits in *Synsepalum dulcificum*

Among the respondents, 94.60% expressed the desire to have a variety/an improved variety of *S. dulcificum*. Those not desiring any improved variety indicated that an improved variety will not be as “powerful” as the actual landraces, specifically referring to a reduction in the species phytochemical compounds.

In total, 18 breeding traits (Table 5) were mentioned by farmers as of interest in any new/improved variety. Overall, the top five farmer-desired breeding traits were: big fruit size > early fruiting > high fruit yielding > dwarf tree > high fruit miraculin content. We recorded one women-specific preferred trait, that was, “uniformity of fruit ripening”, and four men-specific preferred traits, which included: “extended on-tree fruit shelf life”, “high fruit edible ratio”, “low fruit shedding”, and “long biological productivity period”.

**Table 5:** Desired breeding traits by Benin and Ghana farmers for an improved variety of *Synsepalum dulcificum*

Traits	Cumulated score	Rank	Reasons (relative citation frequency)
Big fruit size	202	1st	For more clients and an easy selling (0.57); a better use efficiency due to higher pulp mass (0.19); for more weight for more income (0.19); for performant offsprings (0.03); easy harvesting (0.02)
Early fruiting	191	2nd	Early benefice (consumption, sales) from the species (1)
High fruit yielding	166	3rd	For more income (0.82); for a diversity of utilization (0.18)
Dwarf tree	83	4th	Easy harvesting (0.92); easier integration in agroforestry systems (0.2)
High fruit miraculin content	69	5th	Attract more clients (0.63); landmark characteristics necessary to enjoy the fruit (0.37)
Fruit freshness	53	6th	To attract the buyer/consumer (0.69); for safety of consumption (0.31)
Large tree crown	31	7th	High productivity (0.56); fruit less exposed to stealing (0.1); provide shelter to rest (0.23); easy management operation (0.06); escaping children disturbance (0.05)
Long fruit shelf life	22	8th	Increased storability for a staggered utilization/commercialization (1)
Biotic stress resistance	18	9th	Healthy and productive tree (0.57); longevity of the tree (0.25); to have pest-free and clean fruits (0.18)
Fast growing	15	10th	Reduced waiting time to fruiting (1)
High fruiting frequency	12	11th	For more revenue (0.55); for more fruits at the end of the year (0.36); for permanent availability of the fruits (0.09)
Abiotic stress tolerance	7	12th	Maintenance of fruiting performance (0.68); better water use efficiency (0.16); high survival rate (0.16)
Big stem diameter	7	13th	Higher resistance to abiotic stress (0.57); high production (0.28); withstanding children disturbance (0.15)
Extended on-tree fruit shelf life	7	14th	For a reduced on-farm post ripening loss (1)
Long lasting production	5	15th	For a long-lasting benefit from it (1)
Low shedding	4	16th	For a high production (1)
High fruit edible ratio	2	17th	For more juice from the fruit (1)
Uniformity of ripening	1	18th	For a one-once grouped harvest (1)

The general multi-group similarity indices for farmer-desired breeding traits across the three ecological zones and six sociolinguistic groups were ${\mathrm{C}}_{\mathrm{S}}^3=0.84$ and ${\mathrm{C}}_{\mathrm{S}}^6=0.94$, respectively. Regarding the ecological zones, the Deciduous forest and Guineo-Congolian ecological zones exhibited a higher similarity for farmer-desired breeding traits (${\mathrm{C}}_{\mathrm{S}}^2=0.94$) than any other pair of ecological zones (${\mathrm{C}}_{\mathrm{S}}^2=0.56$ for the pair Guineo-Congolian and Evergreen forest zones; ${\mathrm{C}}_{\mathrm{S}}^2=0.61$ for the pair Deciduous forest and Evergreen forest zones). Likewise, the highest pairwise similarity index between sociolinguistics groups was obtained for *Akan-Ewe*$\left({\mathrm{C}}_{\mathrm{S}}^2=0.96\right)$ and the lowest one for *Ga-adangbe-Sahouè* $\left({\mathrm{C}}_{\mathrm{S}}^2=0.74\right)$(Table 6).

**Table 6:** Similarity of farmer-desired breeding traits for *S. dulcificum* across sociolinguistic groups. Values in the lower diagonal are pair-wise similarity indices of desired traits and those in the upper diagonal are Kendall-W concordance coefficients for traits ranking

	Adja	Fon	Sahouè	Akan	Ewe	Ga-adangbe
Adja		0.97	0.93	0.60	1.00	0.90
Fon	0.84		0.97	0.65	0.97	0.91
Sahouè	0.82	0.74		0.70	0.90	0.70
Akan	0.86	0.78	0.77		0.75	0.70
Ewe	0.89	0.81	0.86	0.96		0.90
Ga-adangbe	0.84	0.75	0.74	0.85	0.88	

Respondents in Guineo-Congolian and Deciduous forest ecological zones exhibited a perfect agreement in the ranking pattern of their desired traits (Kendall-W = 1.00). For respondents of these two ecological zones, big fruit size, early fruiting and high fruit yielding were the top three desired traits for an improved variety of *S. dulcificum*, whereas their counterparts of Evergreen forest ecological zone, instead targeted resistance to biotic stresses, early fruiting, and high fruit yielding as traits of interest (Table 7).

**Table 7:** Ecological zone-based variation of the top five farmer-desired breeding traits for an improved variety of *Synsepalum dulcificum*

Traits	Guineo-Congolian zone		Deciduous forest zone		Evergreen forest zone	
	Cumulated score	Rank	Cumulated score	Rank	Cumulated score	Rank
Big fruit size	78	1st	121	1st	2	4th
Early fruiting	73	2nd	113	2nd	5	1st
High fruit yielding	51	3rd	111	3rd	4	3rd
High fruit miraculin content	26	–	63	4th	–	–
Dwarf tree	26	4th	55	5th	2	4th
Biotic stress resistance		–	–	–	5	1st
Large tree crown	17	5th	–	–	–	–
Overall ranking agreement	Kendall-W = 0.6					

Traits such as big fruit size, early fruiting, high fruit yielding and dwarf tree were consistently ranked among the first four most important farmer-desired breeding traits (Table 8) by the sociolinguistic groups of Benin i.e. *Adja*, *Fon* and *Sahouè*. These sociolinguistic groups also exhibited a very high agreement in their ranking pattern (Kendall-W > 0.90, Table 6). In contrast, a lower agreement was observed among Ghana sociolinguistic groups, the maximum concordance coefficient of desired-traits was 0.90 (Table 6). For instance, for *Ewe* and *Ga-adangbe*, big fruit size was the most important trait desired for an improved variety, while their counterpart A*kan* rather targeted a high fruit yielding variety (Table 8).

**Table 8:** Sociolinguistic group-based variation of the top five farmer-desired breeding traits for an improved variety of *Synsepalum dulcificum*

Traits	Adja		Fon		Sahouè		Akan		Ewe		Ga-adangbe	
	Cumulated score	Rank	Cumulated score	Rank	Cumulated score	Rank	Cumulated score	Rank	Cumulated score	Rank	Cumulated score	Rank
Big fruit size	28	1st	27	1st	23	2nd	38	3rd	42	1st	43	1st
Early fruiting	20	2nd	27	1st	26	1st	42	2nd	38	2nd	38	3rd
High fruit yielding	18	3rd	21	3rd	12	3rd	43	1st	32	3rd	40	2nd
Dwarf tree	14	4th	6	4th	6	4th	18	4th	20	5th	19	4th
Large tree crown	7	5th	–		6	4th	–	–	–	–	–	–
Fruit freshness	–		5	5th	–	–	–	–	–	–	18	5th
High fruit miraculin content	–		–	–	–	–	17	5th	31	4th	–	–
Overall ranking agreement	Kendall-W = 0.69											

#### Local consumers’ preferences for breeding traits in *Synsepalum dulcificum*

The 300-interviewed miracle berry final consumers in Benin and Ghana together listed a total of nine desired traits for an improved variety adapted for consumption. Out of these traits only five, which also represented the top five desired traits have a citation frequency higher than 5% (Table 9). Overall, desired traits were 94.56% similar across the three ecological zones and 83.60% identical across the six sociolinguistic groups. The highest pairwise similarity index of final consumers’ desired breeding traits was obtained between respondents of Deciduous forest and Guineo-Congolian zones (${\mathrm{C}}_{\mathrm{S}}^2=0.87$), while the highest agreement in traits ranking pattern was observed between respondents from Deciduous forest and Evergreen forest ecological zones (Kendall-W = 0.90). The lowest agreement coefficient (Kendall-W = 0.40) of these traits ranking was also observed between final consumers of Guineo-Congolian and Evergreen forest ecological zones. Regarding the sociolinguistic groups, *Adja* and *Sahouè* final consumers preferred similar traits, while *Adja* and *Ga-adangbe* final consumers, on one hand, and *Sahouè* and *Ga-adangbe* final consumers, on the other hand, exhibited the most divergent preference for desired breeding traits (Table 10).

**Table 9:** Final consumer-desired breeding traits for an improved variety of *Synsepalum dulcificum*

Traits	Cumulated score	Rank	Reasons (Citation frequency)
High miraculin content	368	1st	To enjoy the fruit (1)
Big fruit size	350	2nd	For a quick satisfaction (0.74), to attract consumers (0.26)
Fruit freshness	293	3rd	To attract consumer (0.64), for safety of consumption (0.36)
Long fruit shelf life	91	4th	Better storability for staggered consumption over the time (1)
High fruit edible ratio	48	5th	More juice from the fruit (1)
Fruit colour sharpness	41	6th	To make the fruit more attractive for the consumer (1)
Low potency	8	7th	To enjoy the taste of other foods after the consumption of miracle berry (1)
Firmness	6	8th	For better storability (1)
Fruit shape	1	9th	Determined the fruit attractivity (1)

**Table 10:** Pair-wise similarity index across sociolinguistic groups for all consumer-preferred traits for an improved variety of *Synsepalum dulcificum.* Values in the lower diagonal are pair-wise similarity indices of desired-traits and those in the upper diagonal are Kendall-W concordance coefficients for traits ranking

	Adja	Fon	Sahouè	Akan	Ewe	Ga-adangbe
Adja		1.00	0.76	0.70	0.90	0.90
Fon	0.92		0.97	0.70	0.90	0.90
Sahouè	1.00	0.92		0.65	0.81	0.81
Akan	0.76	0.85	0.76		0.90	0.95
Ewe	0.85	0.80	0.85	0.80		1.00
Ga-adangbe	0.66	0.769	0.66	0.92	0.71	

Based on the ecological zone, all final consumers concurred on “big fruit size”, “high fruit miraculin content” and “fruit freshness” as the three most desired traits to consider in breeding an improved miracle berry variety (Table 11). Fruit shape and low potency were the two “men-specific” traits recorded in this study. All the final consumers belonging to Benin’s sociolinguistic groups ranked big fruit size as the most desired trait in an improved miracle berry variety, whereas the Ghanaian sociolinguistic groups indicated “high fruit miraculin content” as their most desired trait in a variety of *S. dulcificum* (Table 12). Nevertheless, the final consumers of five out of the six sociolinguistic groups investigated in this study ranked “big fruit size”, “high fruit miraculin content” and “fruit freshness” as their top three preferred traits for an ideal variety of miracle plant. This was reflected in the high overall concordance coefficient of 0.78 (Table 12).

**Table 11:** Ecological zone**-**based variation of the top five consumer-desired breeding traits for an improved variety of *Synsepalum dulcificum*

Traits	Guineo-Congolian zone		Deciduous forest zone		Evergreen forest zone	
	Cumulated score	Rank	Cumulated score	Rank	Cumulated score	Rank
Big fruit size	134	1st	211	3rd	5	3rd
High fruit miraculin content	78	2nd	284	1st	6	2nd
Fruit freshness	57	3rd	229	2nd	7	1st
High fruit edible ratio	41	4th				
Long fruit shelf life	36	5th	50	4th	5	3rd
Fruit colour sharpness	–	–	5	5th	–	–
Fruit firmness	–	–			2	5th
Overall ranking agreement	Kendall-W = 0.56

**Table 12:** Sociolinguistic group-based variation of the top five consumer-desired breeding traits for an improved variety of *Synsepalum dulcificum*

Traits	Adja		Fon		Sahouè		Akan		Ewe		Ga-adangbe	
	Cumulated score	Rank	Cumulated score	Rank	Cumulated score	Rank	Cumulated score	Rank	Cumulated score	Rank	Cumulated score	Rank
Big fruit size	49	1st	51	1st	34	1st	57	3rd	85	2nd	74	2nd
High fruit miraculin content	22	2nd	41	2nd	15	3rd	104	1st	109	1st	77	1st
Fruit freshness	17	3rd	25	3rd	15	3rd	92	2nd	71	3rd	73	3rd
High fruit edible ratio	11	4th	-	-	18	2nd	-	-	-	-	-	-
Long fruit shelf life	11	4th	13	5th	12	5th	24	4th	13	4th	19	4th
Colour sharpness	–	–	20	4th	–	–	–	–	5	5th	–	–
Low potency	–	–	–	–	–	–	3	5th	–	–	2	5th
Overall ranking agreement	Kendall-W = 0.78											

#### Processor-desired breeding traits in *Synsepalum dulcificum*

At the end of the focus group discussion held with sections' managers from the Miracle Fruit Processing Ghana Ltd company, a total of five main preferred breeding traits were listed in order of importance as: “high fruit miraculin content”, “big fruit size”, “high fruit edible ratio” and “fruit freshness” (for the miracle fruit powder production line) and “high seed portion” (for the dry seed production line).

#### Consistency among end-users’ desired breeding traits in *Synsepalum dulcificum*

The consistency analysis of the top five desired breeding traits among farmers, final consumers and industrial processors (Table 13) revealed a good level of similarity (${\mathrm{C}}_S^3=0.60$) among the three end-user groups although agreement in ranking of these desired traits was in general very low (Kendall-W = 0.11). However, the pairwise similarity index between these three end-user groups revealed that final consumers’ and industrial processors’ top five desired breeding traits were highly similar (80% similarity index), and almost in perfect agreement on the ranking pattern (Kendall-W = 0.90). Conversely, desired breeding traits for an improved variety were divergent from farmers and final consumers, on one hand, to farmers and processors, on the other hand. Either way, the most important traits for all three groups included big fruit size and high fruit miraculin content.

**Table 13:** Consistency parameters among miracle berry end-users for preferred breeding traits in *Synsepalum dulcificum* in Benin and Ghana

**End-users’ preferred breeding traits and ranking**			**Rank**	**Overall ranking agreement**
**Farmers**	**Processors**	**Consumers**		
Big fruit size	High fruit miraculin content	High fruit miraculin content	1st	0.11
Early fruiting	Big fruit size	Big fruit size	2nd	
High fruit yielding	High fruit edible ratio	Fruit freshness	3rd	
Dwarf tree	Fruit freshness	Long fruit shelf life	4th	
High fruit miraculin content	High seed portion	High fruit edible ratio	5th	
**Pair-wise similarity index (lower diagonal) and ranking concordance coefficient (upper diagonal) of preferred trait between end-user groups**				
	**Farmers**	**Processors**	**Consumers**	
Farmers		0.00	0.00	
Processors	0.40		0.90	
Consumers	0.40	0.80		

## Discussion

### Respondents socio-demographic profile, tree holding system and relative importance of *Synsepalum dulcificum* in current production systems

Men were the main respondents in this study as they were the most involved in *S. dulcificum* management. This comes without surprise as it is known that management of perennial species is mainly carried out by men [49]. Indeed, planting perennial species is culturally bound with land ownership [50] and it is true that in West Africa, men are the prominent landowners [51]. For instance, in this study, men had on average 9.16 ± 2.77 ha, whereas women only had 2.53 ± 0.03 ha. However, the more frequent involvement of men in *S. dulcificum* management is not translated into a higher number of trees held compared with women. This finding aligned with that of Fandohan et al. [6] who did not detect any differences in the number of *S. dulcificum* trees possessed by men and women in Benin, but is contrary to observation in other perennial species [e.g. bitter kola (*Garcinia kola* Heckel)] [52] where men owned more trees than women. This implies that if given the opportunity, women can contribute equally as men to conserve and manage *S. dulcificum*, as their management intensity index is also statistically comparable to that of men in this study.

Our findings also corroborated results of Fandohan et al. [6] on the variation in the number of trees possessed following sociolinguistic group affiliation. Moreover, we detected an ecological gradient signal in the number of trees possessed, with respondents in drier ecological zones owning fewer trees compared with those in moister ecological zones. This confirms the crucial role of water availability and rainfall in the establishment and development of the species [53]. We could also speculate that the more market-oriented production in Ghana (moister ecological zones than in Benin) compared with Benin where the market is still emerging could have provided incentives for Ghanaian farmers to protect/grow more trees, though less than 5% have commercial plantations.

Crops listed by respondents as being important in their production system mostly included those already well established due to their high potential to contribute to food security (e.g. maize, cassava [54]) and substantial income/cash generation (e.g. cocoa, coffee [55]) or to stand as multi-purpose species (e.g. palm oil combining high market and food values). Yet, it is found in this study that miracle berry could locally generate up to USD 893.76 as seasonal income while maintaining a high market value internationally with nearly USD 2500/kg of pure powder (https://www.miraclefruitfarm.com/)*.* The poor ranking of the species despite its staggering market value might be explained by its low caloric value coupled with an incapacity of the current production system to expose the crop potential. As revealed by our findings, only a low proportion of respondents currently have the species in plantation while plantation size is still low. Then, it appears necessary to increase awareness of *S. dulcificum* potential to encourage more producers to meet the growing demand for the species. To that, unravelling socio-economic, biological and cultural drivers for sustainable cultivation of the species is required.

### *Synsepalum dulcificum* management practices

Quality planting material is crucial for a successful establishment and subsequent development of tree species [56]. In this study, although seeds and seedlings were co-used by respondents, most of them established their trees/plantations using seedlings. This preference for seedlings transplanting was also reported in *B. sapida* [23] and *G. kola* [52] two other minor orphan tree crops, and partly appeared in the case of *S. dulcificum* as a strategy to skirt the difficulty to germinate its recalcitrant seeds [4]. According to farmers, using the seed compels one to quickly sow it, which is not the case with seedlings that have been harvested beneath *S. dulcificum* plants or orchards of which planting can be postponed and carried out later. Besides, using seedlings offered the advantage to select highly vigorous individuals, thus ensuring better growth and productivity. This could be the main reason why in the Evergreen forest ecological zone, which is dominated by plantations of *S. dulcificum*, respondents mainly used seedlings, sourced from the Plant Genetic Resources Research Institute, Bunso (a national research centre previously interested in promoting *S. dulcificum* in Ghana). As also observed in other crops such as *Oryza sativa* L. [57], *Solanum tuberosum* L. [58], *M. esculenta* [27] and *M. geocarpum* [17], exchange of plant materials prevailed in *S. dulcificum* among farmers and in all sociolinguistic groups, with sometimes the exchange taking place between farmers from distant districts. Contrary to observations in cassava where planting material exchange between farmers is partly through monetary means [27], planting material exchange in the case of *S. dulcificum* occurred only as a gift, probably because most of the respondents perceived the species as not important in their production system*.* The quasi-absence or inactivity of a research centre focusing on the crop could have also favoured the magnitude of landraces exchanged among farmers. However, the *Adja* and *Fon* were the only farmers who accessed planting materials from the market. This could result from them being the only two sociolinguistic groups that transact the fruit on local markets.

Our results revealed that management intensity varied following ecological conditions and sociolinguistic groups as reported by Blancas et al. [47], but also following farmer’s instruction level. Overall, management practices are easier and more rigorously applied when environmental conditions are conducive. As indicated above, the Evergreen forest ecological zone has a very high annual rainfall, which is favourable to the species. Besides respondents in that ecological zone being in contact with a research centre for the acquisition of planting materials, they could have also likely benefited from technical advice enabling them to accumulate knowledge to apply more rigorously management practices. Indeed, specific practices such as pegging, lining and pruning were applied by 60–100% of respondents in the Evergreen zone against only 11.5–26.1% of those in Deciduous forest and Guineo-Congolian zones. Furthermore, being educated offers a competitive advantage in terms of understanding potential, ability to take initiative, to innovate and to try new technologies [15, 59]. This might explain why the educated respondents in general exerted a higher management intensity. The *Akan* represented the only sociolinguistic group that owned plantations; and maintaining a high level of productivity in these plantations made it necessary for them to exert a higher maintenance effort, which would have translated into the highest management intensity index observed in this sociolinguistic group.

The lack of improved *S. dulcificum* variety confirmed the absence of breeding programmes for the crop in West Africa. In such a context, farmer’s knowledge represents a good asset to establish a sound and sustainable one. Indeed, farmers’ knowledge of morphological variation in the species is in line with literature reports. For instance, the small fruit- and big fruit-sized morphotypes distinguished by the farmers in this study were previously reported by Coronel et al. [60].

### End-users’ preferences

This study highlighted a diversity of traits desired by *S. dulcificum*’s end-users and revealed that farmers desired more breeding traits than final consumers, while the farmers and final consumers combined desired more traits than industrial processors. This indicated a specialization tendency for trait preference along the species value-chain. Within either farmers’ or consumers’ groups, the high similarity of desired traits across not only ecological zones but also sociolinguistic groups implied that a unique variety will work for each group at the level of the West African sub-region. More importantly, both final consumers and farmers in ecologically closer zones tended to have a more similar preference for breeding traits. The trend in final consumer-desired breeding traits aligned with the ethnocentrism hypothesis which predicts that ethnically close final consumers exhibited a similar choice pattern towards specific produces [61].

It is reported that farmers ranked their preference for breeding traits to meet different scenarios including the necessity to mitigate or overcome their production constraints [62]. Here, the most important farmer-desired traits in *S. dulcificum* were towards ensuring a high profitability. Indeed, early fruiting reduces waiting time and ensures a quick acquisition of the marketable produce —the fruit—which is better harvested on short trees and whose size conditioned the yield, this latter together with the taste (miraculin content) being determinant in attracting clients. Fruit tree species have a long juvenility and early fruiting is a strongly desired trait for all of them. Bhargava and Srivastava [63] argued that early fruiting also helps reduce management period and cost and thus increases profitability. As reported in *M. esculenta* [27] and *Musa* spp. [62], gender-specific desired traits also exist in *S. dulcificum* and seemed to illustrate gender role in the species’ value chain. For instance, the “uniformity of ripening” was only mentioned by women who are known as the main harvesters/collectors and sellers of the fruit on local markets. For them, a uniform ripening could ensure a one-once and cost-efficient harvest as well as a grouped selling. Contrary to women, men do not sell the fruit in markets, but rather wait for retailers, processors or final consumers to come to them, hence their desire to have the ripe fruits to be on the trees over a longer period. This justifies the importance of the trait “extended on-tree fruit shelf life” that is specific to men. The significance of some traits poorly ranked today might increase in the future especially when it will come to large-scale cultivation. This is the case of the uniformity or ripening, a trait deemed of paramount importance in mechanized fruit harvesting [64].

For final consumers, a preferred variety of *S. dulcificum* should encompass traits that will contribute to attracting them and maximizing their satisfaction. Ahead of such traits is the miraculin content, driver of the landmark attribute of the miracle berry, that is, its ability to induce sweetness. This creates an impetus for exploitation of miraculin coupled with the fruit size and the fruit freshness. As indicated by our results, the ranking of these three traits at the overall level also corroborates their ranking across ecological zones and sociolinguistic groups, thus strengthening their high importance for final consumers.

## Implications for future research and breeding

The moderate similarity of breeding traits among the three end-user groups suggested the necessity to foresee variety development per functionally similar group. Consequently, in the light of pairwise-similarity index obtained in this study, final consumers and industrial processing could be targeted when developing a specific variety and farmers exclusively when developing a different one. However, the three groups of end-users shared the most valuable desired traits in the species which include big fruit size and high fruit miraculin content.

Breeding perennial plant species is a long-lasting and demanding process in which each step is to be carefully conducted. In the case of *S. dulcificum*, the clear identification of end-users’ preferred traits at the beginning of the process constitutes a robust basis for subsequent steps. In this study, we identified the key traits that can be incorporated in any breeding programme targeting the species improvement in West Africa. In absence of improved varieties in the species, the first necessity is to develop materials that significantly outperform current landraces for the elicited traits. This requires the proper evaluation of the existing diversity for an informed choice of parental lines to be advanced. *Synsepalum dulcificum* being a tree species, the very first step in this attempt is to identify potential elite trees, taking into consideration end-users’ preferred traits and an in situ phenotypic characterization of the species in its centre of diversity could offer first insights. Besides, information on how end-user-desired traits are correlated could inform on the relevance of the development of index selection for parallel and multi-traits selection. Furthermore, a genome-wide scan of the phenotypically characterized groups will help establish sound breeding populations towards varietal development and ensure a sustainable management of the remaining genetic resource diversity. Given its perennial nature, *S. dulcificum* will also benefit from the implementation of genomic selection as a chief- approach in selection strategies.

## Conclusion

This study is the first of its kind to analyse management practices and breeding traits preference by end-users in *S. dulcificum*. Our findings provide first-order information that will feed the on-going pre-breeding process of the miracle berry in West Africa. From the holding system to the revenue generated by the crop to major end-user’s preferred traits, this study compiled important information that will contribute to approaches to develop improved varieties and to enhance the crop’s value chain.

In particular, we found that management practices were relatively more advanced in the Evergreen forest ecological zone than in the two other ecological zones of the study, with the *Akan* applying more rigorously ideal practices. While individual tree ownership was, in general, the dominant holding system of the species in West Africa, a prominence of plantations was observed in the Evergreen zones of Ghana. *Synsepalum dulcificum* has the potential to become one of the main cash crops in West Africa; but for this to be effective, development of improved material for sustainable cultivation is desired. The most influential traits desired by end-users include early fruiting, high fruit yielding, big fruit size, long fruit shelf-life, tree dwarfism, high fruit edible ratio, high fruit miraculin content and fruit freshness. Consequently, any breeding programmes targeting the species’ improvement in West Africa should prioritize these traits to meet multi-actor expectations. Because *S. dulcificum* is currently poorly cultivated, there is a necessity to promote/value the crop and explore factors that serve as drivers to trigger its sustainable cultivation in West Africa in order to combine the preservation of a fragile biodiverse environment with the growing demand and enable grass-root populations to benefit from the species potential.

## Supplementary Information

**Additional file 1: Supplementary Table S1.** Socio-demographic profile of individual respondents. **Supplementary Table S2.** Complete list of plant species recorded in production systems involving *Synsepalum dulcificum* in the study area. **Supplementary Table S3.** Per gender-disaggregated importance of species involved in *Synsepalum dulcificum*-based production system.

**Additional file 2. **Sociolinguistic group-based disaggregation of top five important species in *Synsepalum dulcificum*-based production system.

## Data Availability

The datasets supporting the conclusions of this article are included within the article (and its additional files).

## Abbreviations

BMI: Boosted management intensity index
DF: Deciduous forest ecological zone
EF: Evergreen forest ecological
GC: Guineo-Congolian zone
USA: United States of America
