# Supplementary material for: Comparative analysis of management practices and end-users’ desired breeding traits in the miracle plant [Synsepalum dulcificum (Schumach & Thonn.) Daniell] across ecological zones and sociolinguistic groups in West Africa
Source: J Ethnobiol Ethnomed. 2021 Jun 19;17:41. doi: 10.1186/s13002-021-00467-8 (PMC8214305; doi:10.1186/s13002-021-00467-8)
Supplement: Supplementary file 1 — Additional file 1: Supplementary Table S1. Socio-demographic profile of individual respondents. Supplementary Table S2. Complete list of plant species recorded in production systems involving Synsepalum dulcificum in the study area. Supplementary Table S3. Per gender-disaggregated importance of species involved in Synsepalum dulcificum-based production system. [file 13002_2021_467_MOESM1_ESM.docx]

**Comparative analysis of management practices and end-users’ desired breeding traits in the miracle plant [*Synsepalum dulcificum* (Schumach & Thonn.) Daniell] across ecological zones and sociolinguistic groups in West Africa**

Dèdéou A. Tchokponhoué^1,2^*, Enoch G. Achigan-Dako^2^, Sognigbé N’Danikou^2,3^, Daniel Nyadanu^4^, Rémi Kahane^5^, Alfred O. Odindo^1^ and Julia Sibiya^1^

^1^School of Agricultural, Earth and Environmental Sciences, University of KwaZulu-Natal, Private Bag X01, Scottsville, 3209, Pietermaritzburg, South Africa.

^2^Laboratory of Genetics, Biotechnology and Seed Science (GBioS), School of Plant Sciences, University of Abomey-Calavi, 01 BP 526, Abomey-Calavi, Republic of Benin.

^3^World Vegetable Center, East and Southern Africa, Po. Box 10 Duluti, Arusha, Tanzania

^4^Cocoa Research Institute of Ghana (CRIG), P. O. Box 8, Akim Tafo, Ghana.

^5^Research Unit HortSys, Department Persyst, CIRAD, Campus de Baillarguet, 34398 Montpellier cedex 5, France.

*** Corresponding author:** Dèdéou A. Tchokponhoué

Email: [dedeoutchokponhoue@gmail.com](mailto:dedeoutchokponhoue@gmail.com)

**Supplementary Table S1**, **additional file 1** Socio-demographic profile of individual respondents

| Country | | Benin | | | Ghana | | | Total  N = 300 |
| --- | --- | --- | --- | --- | --- | --- | --- | --- |
| Ecological zone | | Guineo-Congolian | | | Deciduous + Evergreen forest | Deciduous forest | |  |
| Sociolinguistic group | | Adja  (n = 46) | Fon  (n = 62) | Sahouè  (n = 38) | Akan  (n = 52) | Ewe  (n = 51) | Ga-adangbe  (n = 51) |  |
| Gender | Female (%) | 1.33 | 3.00 | 1.67 | 2.67 | 2.67 | 2.33 | 13.67 |
|  | Male (%) | 14.00 | 17.66 | 11.00 | 14.67 | 14.33 | 14.67 | 86.33 |
| Instruction level | Illiterate (%) | 8.00 | 10.67 | 6.33 | 1.00 | 0.67 | 3.67 | 30.34 |
|  | Literate (%) | 1.00 | 0.67 | 0.00 | 0.67 | 0.00 | 0.33 | 2.67 |
|  | Primary (%) | 3.00 | 5.33 | 3.33 | 2.66 | 3.00 | 4.67 | 21.99 |
|  | Secondary (%) | 3.33 | 3.00 | 2.33 | 11.33 | 11.67 | 8.00 | 39.66 |
|  | ≥ BAC (%) | 0.00 | 1.00 | 0.67 | 1.67 | 1.67 | 0.33 | 5.34 |
| Marital status | Single (%) | 0.33 | 0.33 | 0.33 | 0.67 | 2.00 | 1.33 | 4.99 |
|  | Married (%) | 15.00 | 18.33 | 11.67 | 14.00 | 13.33 | 12.67 | 85.00 |
|  | Widower (%) | 0.00 | 2.00 | 0.67 | 2.67 | 1.67 | 3.00 | 10.01 |
| Migration | Autochtons (%) | 14.33 | 18.67 | 12.67 | 14.67 | 15.66 | 16.00 | 92.00 |
|  | Allocthons  (%) | 1.00 | 2.00 | 0.00 | 2.67 | 1.33 | 1.00 | 08.00 |
| Age (year) | Range | 25 - 88 | 26 - 89 | 30 - 85 | 29 - 83 | 26 - 87 | 30 - 102 | 25 - 102 |
|  | Mean ± se | 54.46 ± 2.31 | 52.10 ± 2.16 | 57.95 ± 2.72 | 54.65 ± 1.91 | 56.90 ± 2.06 | 58.11 ± 2.55 | 56 .00 ± 01 |
| Landholding (ha) | Range | 0.40 - 25 | 0.04 - 30 | 0.40 - 40.00 | 0.16 - 3.51 | 0.40 - 7.02 | 0.80- 16.18 | 0.40 - 7.02 |
|  | Mean ± se | 3.16 ± 0.59 | 3.42 ± 0.57 | 4.74 ± 1.15 | 13.33 ± 5.92 | 20.13 ± 2.63 | 4.35 ± 0.39 | 8.14 ± 2.41 |
| Household size | Range | 01 - 30 | 01 - 16 | 02 - 19 | 01 - 20 | 01 - 13 | 0 - 19 | 00 - 30 |
|  | Mean ± se | 10.70 ± 0.98 | 6.55 ± 0.39 | 7.95 ± 0.58 | 06.42 ± 0.45 | 5.92 ± 0.41 | 6.63 ± 0.49 | 07.25 ± 0.24 |

**Supplementary Table S2, Additional file 1** Complete list of plant species recorded in production systems involving Synsepalum dulcificum in the study area

| Botanical family | Species (**Voucher specimen code**) | Production systems |
| --- | --- | --- |
| Anacardiaceae | *Mangifera indica* L. (**DAT01**) | Home-garden, farm |
| Annonaceae | *Annona muricata* L. (**DAT13**) | Home-garden, farm |
|  | *Monodora myristica* (Gaetn.) Dunal (**DAT11**) | Home-garden, farm |
|  | *Xylopia aethiopica* (Dunal) A. Rich. (**DAT04**) | Home-garden, farm |
| Apocynaceae | *Picralima nitida* (Stapf.) T.& H.Durand (**DAT30**) | Home-garden, farm |
| Araceae | *Colocasia esculenta* (L.) Schott (**DAT16**) | Home-garden, farm |
| Arecaceae | *Borassus aethiopum* Mart. (**DAT07**) | Home-garden |
|  | *Cocos nucifera* L. (**DAT08**) | Home-garden, farm |
|  | *Elaeis guineensis* Jacq. (**DAT09**) | Home-garden, farm |
| Asteraceae | *Vernonia amygdalina* Delile (**DAT10**) | Home-garden |
| Bignoniaceae | *Crescentia cujete L.* (**DAT03**) | Home-garden |
|  | *Newbouldia laevis* (P.Beauv.) Seem (**DAT12**) | Home-garden |
| Brassicaceae | *Brassica oleracea L.* (**DAT02**) | Farm |
| Bromeliaceae | *Ananas comosus* (L.) Merr. (**DAT19**) | Home-garden, farm |
| Caricaceae | *Carica papaya* L. (**DAT14**) | Home-garden, farm |
| Clusiaceae | *Garcinia kola* Heckel (**DAT05**) | Home-garden, farm |
| Combretaceae | *Anogeissus leiocarpa* (DC.) Guill. & Perr. (**DAT06**) | Home-garden |
|  | *Terminalia superba* Engl. & Diels (**DAT17**) | Farm |
| Dioscoreaceae | *Dioscorea alata* L. (**DAT21**) | Home-garden, farm |
|  | *Discorea dumetorum* (Kunth) Pax (**DAT20**) | Farm |
| Euphorbiaceae | *Hevea brasiliensis* (Willd. Ex A. Juss.) Mull.Arg. (**DAT18**) | Farm |
|  | *Manihot esculenta* Crantz. (**DAT22**) | Home-garden, farm |
|  | *Manihot glaziovii* Mull. Arg. (**DAT35**) | Home-garden |
| Irvingiaceae | *Irvingia gabonensis* (Aubry-Lecomte ex O'Rorke) Baill. (**DAT24**) | Home-garden, farm |
| Lamiaceae | *Tectona grandis* L.f. (**DAT27**) | Home-garden, farm |
| Lauraceae | *Persea americana* Mill. (**DAT37**) | Home-garden, farm |
| Leguminosae | *Acacia auriculiformis* Benth. (**DAT25**) | Farm |
|  | *Arachis hypogaea* L. (**DAT28**) | Home-garden, farm |
|  | *Caesalpinia bonduc* (L.) Roxb. (**DAT29**) | Home-garden |
|  | *Glycine max* (L.) Merr. (**DAT05**) | Farm |
|  | *Phaseolus lunatus* L. (**DAT31**) | Farm |
|  | *Pterocarpus santalinoides* DC. (**DAT32**) | Home-garden |
|  | *Vigna unguiculata* (L.) Walp (**DAT51**) | Home-garden, farm |
| Malvaceae | *Abelmoschus esculentus* (L.) Moench (**DAT34**) | Home-garden, farm |
|  | *Cola nitida* (Vent.) Schott & Endl. (**DAT23**) | Home-garden, farm |
|  | *Theobroma cacao* L. (**DAT36**) | Home-garden, farm |
| Meliaceae | *Azadirachta indica* A.Juss. (**DAT26**) | Home-garden |
|  | *Khaya senegalensis* (Desv.) A. Juss (**DAT38**) | Home-garden, farm |
| Moraceae | *Artocarpus altilis* (Parkinson ex F.A.Zorn) Fosberg (**DAT50**) | Home-garden |
|  | *Milicia excelsa* (Welw.) C.C.Berg (**DAT49**) | Farm |
| Moringacea | *Moringa oleifera* (Gaetn.) Dunal (**DAT41**) | Home-garden |
| Musaceae | *Musa parasidica* L. (**DAT58**) | Home-garden, farm |
|  | *Musa sapientum* L. (**DAT62**) | Home-garden, farm |
| Myrtaceae | *Eucalyptus camaldulensis* Dehnh. (**DAT44**) | Home-garden |
|  | *Psidium guajava* L. (**DAT45**) | Home-garden, farm |
| Oxalidaceae | *Averoa carrambola* L. (**DAT52**) | Home-garden |
| Passifloraceae | *Passiflora edulis* Sims (**DAT47**) | Farm |
| Poaceae | *Bambusa vulgaris* Schrad. (**DAT48**) | Home-garden |
|  | *Saccharum officinale* L. (**DAT40**) | Home-garden |
|  | *Zea mays* L. (**DAT39**) | Home-garden, farm |
| Rubiaceae | *Coffea canephora* L. (**DAT53**) | Home-garden, farm |
| Rutaceae | *Citrus limon* L. (**DAT46**) | Home-garden, farm |
|  | *Citrus sinensis* (L.) Osbeck (**DAT33**) | Home-garden, farm |
| Sapotaceae | *Chrysophyllum albidum* G.Don (**DAT60**) | Home-garden, farm |
|  | *Synsepalum dulcificum* (Schumach & Thonn.) Daniell (**DAT55**) | Home-garden, farm |
| Solanaceae | *Capsicum spp* (**DAT56**) | Home-garden, farm |
|  | *Lycopersicum esculentum L.* (**DAT57**) | Home-garden, farm |
|  | *Solanum aethiopicum* L. (**DAT42**) | Home-garden, farm |
|  | *Solanum macrocarpon* L. (**DAT59**) | Home-garden, farm |
|  | *Solanum melongena* L. (**DAT54**) | Home-garden |
| Zingiberaceae | *Afromomum melegueta* K.Schum. (**DAT61**) | Farm |
|  | *Zingiber officinale* Roscoe (**DAT43**) | Farm |

**Supplementary Table S3, Additional file 1** Per gender-disaggregated importance of species involved in S. dulcificum-based production systems

| Production system | Men | | | Women | | |
| --- | --- | --- | --- | --- | --- | --- |
|  | Species | IS* | Rank | Species | IS* | Rank |
| Farm | *Theobroma cacao* L. | 4.09 | 1^st^ | *Zea mays* L*.* | 3.88 | 1^st^ |
|  | *Zea mays* L. | 3.98 | 2^nd^ | *Theobroma cacao* L. | 3.77 | 2^nd^ |
|  | *Elaeis guineensis* Jacq. | 3.50 | 3^rd^ | *Manihot esculenta* Crantz. | 3.50 | 3^rd^ |
|  | *Manihot esculenta* Crantz. | 3.30 | 4^th^ | *Elaeis guineensis* Jacq. | 3.09 | 4^th^ |
|  | *Solanum aethiopicum* L. | 3.13 | 5^th^ | *Acacia auriculiformis* Benth. | 3.00 | 5^th^ |
| Home-garden | *Theobroma cacao* L. | 4.35 | 1^st^ | *Theobroma cacao* L. | 4.50 | 1^st^ |
|  | *Elaeis guineensis* Jacq. | 3.75 | 2^nd^ | *Musa parasidica* L. | 4.00 | 2^nd^ |
|  | *Musa parasidica* L. | 3.46 | 3^rd^ | *Persea americana* Mill. | 4.00 | 3^rd^ |
|  | *Musa sapientum* L. | 3.08 | 4^th^ | *Chrysophyllum albidum* G.Don | 3.33 | 4^th^ |
|  | *Cocos nuciferae* L. | 2.85 | 5^th^ | *Colocasia esculenta* (L.) Schott | 3.33 | 5^th^ |

***IS**: Importance score.
